# Comparative Analysis of Diel and Circadian Eclosion Rhythms and Clock Gene Expression Between Sexes in the Migratory Moth *Spodoptera frugiperda*

**DOI:** 10.3390/insects16070705

**Published:** 2025-07-09

**Authors:** Changning Lv, Yibo Ren, Viacheslav V. Krylov, Yumeng Wang, Yuanyuan Li, Weidong Pan, Gao Hu, Fajun Chen, Guijun Wan

**Affiliations:** 1 Sanya Research Institute, Nanjing Agricultural University, Sanya 572025, China; 2022202015@stu.njau.edu.cn (C.L.); 2023102086@stu.njau.edu.cn (Y.R.); yumengwang@njau.edu.cn (Y.W.); hugao@njau.edu.cn (G.H.); fajunchen@njau.edu.cn (F.C.); 2 State Key Laboratory of Agricultural and Forestry Biosecurity, College of Plant Protection, Nanjing Agricultural University, Nanjing 210095, China; 3 Papanin Institute for Biology of Inland Waters of the Russian Academy of Sciences, Borok 152742, Yaroslavl Oblast, Russia; kryloff@ibiw.ru; 4 Faculty of Biology and Health Education, Cherepovets State University, Cherepovets 162600, Vologda Oblast, Russia; 5 Beijing Key Laboratory of Bioelectromagnetics, Institute of Electrical Engineering, Chinese Academy of Sciences, Beijing 100190, China; lyy@mail.iee.ac.cn (Y.L.); panwd@mail.iee.ac.cn (W.P.)

**Keywords:** *Spodoptera frugiperda*, circadian rhythm, eclosion timing, clock gene expression, sex differences, light regime, migratory insect pest

## Abstract

The circadian clock orchestrates behavioral and molecular processes such as eclosion. Understanding eclosion timing may offer insights into circadian mechanisms underlying migratory timing. Here, we characterize the diel and circadian patterns of eclosion and core clock gene expression in the fall armyworm (FAW), *Spodoptera frugiperda*, a globally distributed migratory moth. Using a custom-designed eclosion monitoring system under 14 h light: 10 h dark (L14: D10) and constant darkness (DD) conditions, we observed robust diel eclosion rhythms peaking shortly after lights-off under L14: D10, which became delayed and damped over three consecutive days in DD. Males showed a tendency toward more dispersed emergence patterns and exhibited statistically distinguishable eclosion distributions from females under both conditions. Expression of five canonical clock genes (*cyc*, *clk*, *tim*, *per*, *cry2*) displayed significant 24 h rhythmicity, with generally higher mesors in males. However, sex-specific differences in amplitude and phase were detected only for *clk* and *cyc* under L14: D10, not in DD. These findings suggest that sex-specific differences in circadian regulation are limited. Nonetheless, subtle variations in clock gene output and emergence timing in the FAW population established in China may contribute to sex-specific ecological strategies in the novel migratory arena.

## Simple Summary

Many organisms exhibit circadian rhythms in key behaviors, including the timing of adult emergence. Here, we investigated diel and circadian eclosion rhythms and clock gene expression in female and male fall armyworms (FAW), *Spodoptera frugiperda*, a globally distributed migratory moth species of major agricultural importance that recently invaded China. Using a custom-built monitoring system, we recorded adult eclosion under light–dark (LD) and constant darkness (DD) conditions over multiple days. Moths showed a prominent dusk-peaking emergence pattern under LD, which shifted under DD, confirming the presence of an endogenous rhythm. Eclosion distributions differed slightly between sexes under LD and more significantly under DD, suggesting potential sex-specific circadian regulation. We further analyzed transcript expression patterns of five core clock genes (*cyc*, *clk*, *tim*, *per*, *cry2*) in adult heads. While all genes exhibited circadian oscillations under LD and/or DD, sex differences were primarily observed in mesor levels rather than phase or amplitude. These results indicate that male and female moths may differ subtly in the output of their internal clocks, which could underlie sex-specific behavioral strategies. Our findings contribute to understanding the circadian biology of this invasive species in China and may inform timing-based forecasting and management strategies for FAW.

## 1. Introduction

Circadian timing enables animals to synchronize their physiology and behavior with the daily cycle, allowing them to anticipate and adapt to environmental fluctuations. Circadian rhythms are generated by highly conserved molecular clocks across animals and regulate a wide range of processes [1,2]. While the core architecture of circadian clocks is generally conserved across sexes within a species, sexual dimorphism in circadian systems has emerged as an important topic in chronobiology. Growing evidence indicates that males and females may differ in their circadian behaviors, clock gene expression, and physiological rhythms across multiple species [3,4,5,6,7,8,9,10]. However, the evolutionary and functional significance of these differences remains poorly understood in most insects.

One of the earliest circadian outputs to be studied is the rhythm of adult emergence (eclosion) in holometabolous insects [11,12,13]. Eclosion marks the completion of metamorphosis and is typically gated to dawn or dusk, depending on the species. This event exhibits circadian rhythmicity at the population level, persisting under constant conditions and demonstrating temperature compensation—hallmarks of endogenous circadian control [14,15,16]. Under natural conditions, synchrony between the internal circadian system and external environmental cues such as the photoperiod, the principal zeitgeber, or temporal cue, facilitates the optimal timing of eclosion in a species-specific manner, suggesting that the circadian system restricts emergence to the most ecologically advantageous periods of the day [17]. Recent work in *Drosophila* has shown that the circadian clock gates eclosion by rhythmically modulating the terminal steps of metamorphosis [18,19]. The circadian clock is governed by a conserved transcriptional–translational feedback loop (TTFL) involving core clock genes shared across most insects, including *cycle* (*cyc*), *clock* (*clk*), *timeless* (*tim*), *period* (*per*), and *cryptochrome* (*cry*) [20,21]. Oscillations in the expression of these genes within central and peripheral tissues regulate downstream clock-controlled genes, which in turn orchestrate overt rhythmic behaviors like eclosion. Studies have demonstrated that disruption of these core clock genes alters the timing and rhythmicity of eclosion [22,23,24,25], establishing eclosion as one of the most accessible and ecologically relevant behavioral outputs of the circadian clock. Utilizing eclosion as a model behavior provides a powerful framework for dissecting circadian clock mechanisms and understanding their contributions to insect adaptation [26,27,28,29].

Circadian clocks play a critical role in regulating the physiology and behavior of migratory insects, such as mediating the seasonal induction of migratory traits, regulating flight rhythms, determining reproductive state, and facilitating orientation during migration [27,30,31,32,33]. Precise circadian timing becomes especially important for migrants as individuals must adjust rapidly to shifting photoperiods and environmental cues across broad geographic gradients. Thus, the circadian system may serve as a central temporal framework that integrates endogenous rhythms with environmental signals, enabling migratory insects to time their departure, orientation, and arrival in synchrony with ecological opportunities, although the underlying mechanisms remain to be further elucidated [33]. One such migratory species is *S. frugiperda*, a globally distributed noctuid moth and major agricultural pest. Originating from the tropical and subtropical areas of the Americas, this pest was first detected in the African regions of Nigeria and Ghana in 2016 [34]. Exploiting its formidable migratory capacity, it rapidly expanded its invasive range across Sub-Saharan Africa, the Middle East, much of Asia, and Oceania [35,36]. By December 2018, it had reached Jiangcheng County, Yunnan Province, China [37,38,39]. From year-round breeding zones in South China and Southeast Asia, *S. frugiperda* spreads northward across East Asia during the spring and summer months [40,41,42,43]. Our flight simulation studies revealed that *S. frugiperda* exhibits north-northwest flight orientations in spring and southwest orientations in autumn, consistent with seasonal forward and return migrations across East Asia. Photoperiod has been identified as the principal environmental cue driving this seasonal shift [44]. Recent studies in migratory insects have shown that photoperiod responsiveness is mediated by circadian clock-controlled pathways [26,45,46], suggesting a potential role for the circadian system in the temporal regulation of migratory orientation in *S. frugiperda*.

In our previous work, we established an eclosion rhythm assay for *S. frugiperda* and demonstrated circadian regulation under constant conditions, but the data were based on mixed-sex samples collected during the summer of 2020 [47]. Whether males and females differ in their circadian regulation of eclosion remains unknown. Another clock-related behavior in this species is allochronic mating behavior observed between two morphologically identical but genetically distinct strains, which is a trait unique to native populations in the United States [48,49]. A recent simulation study proposed that sex-dimorphic expression of circadian rhythms could facilitate allochronic speciation by enabling males and females to maintain distinct chronotypes, potentially mitigating the costs of male–male competition [50]. However, empirical support for sex-specific circadian divergence remains lacking, even in the native range. In contrast to native populations, invasive *S. frugiperda* populations in China exhibit a largely homogeneous genetic background distinct from their native counterparts, likely due to hybridization between the strains during the invasion process [51]. This provides a unique opportunity to ask whether sex-specific divergence in circadian traits can arise in *S. frugiperda* in the absence of known strain-level allochrony or reproductive isolation, thereby providing a potential baseline for assessing whether such divergence may precede and potentially facilitate subsequent ecological or reproductive differentiation.

In this study, we used *S. frugiperda* as a model to characterize diel and circadian eclosion rhythms as well as clock gene (*cyc*, *clk*, *tim*, *per*, *cry2*) expression between sexes under summer-like photoperiod and constant darkness conditions. This design allowed us to assess whether any observed sex-related differences reflect intrinsic circadian regulation or are driven by environmental light cues. By focusing on an invasive population lacking known strain-level allochrony, we aimed to explore whether sex-specific divergence in circadian traits could emerge in the absence of established temporal differentiation. This work provides foundational chronobiological data comparing circadian traits between sexes in an invasive population of *S. frugiperda*, providing a reference point for future comparisons across populations and offering empirical context for evaluating theoretical models linking sex-specific circadian expression to allochronic speciation [50]. More broadly, our findings contribute to ongoing efforts to understand the temporal organization of migratory insects and may inform future strategies for their management [52].

## 2. Materials and Methods

### 2.1. Insect Stock

The fall armyworm, *S. frugiperda*, population used in this study was originally collected during the migration season from a maize field in Yuanjiang County, Yuxi City, Yunnan Province, China (23.604° N, 101.977° E) in 2023. Both larval and adult stages were sampled, with ~400 individuals collected in total. All experiments were conducted using the F2 generation reared under laboratory conditions from this field-collected population. They were housed in the laboratory under a summer-like photoperiod of 14 h light: 10 h dark (L14: D10) with light intensity of 1000 lux at 26 ± 1 °C and 70 ± 5% relative humidity (RH), to mimic a summer-like condition when they cause the most crop damage and conduct migration from south to north [38,42]. The first to third instar larvae of *S. frugiperda* were reared with fresh sweet maize leaves (Huajintian No. 2 sweet maize) in plastic containers measuring 40 × 20 × 15 cm. The fourth to sixth instar larvae were placed individually in *Drosophila* vials (2.4 cm bottom diameter, 9.5 cm height) with a sufficient artificial diet [53]. The vials were sterilized, and fresh artificial diets were added every three days to maintain a clean environment and ensure adequate food supply until pupation. The pupation date was recorded, and the sex was identified before the pupae were transferred to new numbered *Drosophila* vials containing a moisturized cotton ball to maintain a relatively moist environment. Adults of *S. frugiperda* were fed using a 10% honey solution daily.

### 2.2. Diel Eclosion Behavior Assay Under the Summer-like L14: D10 Cycle

**Figure 1:**
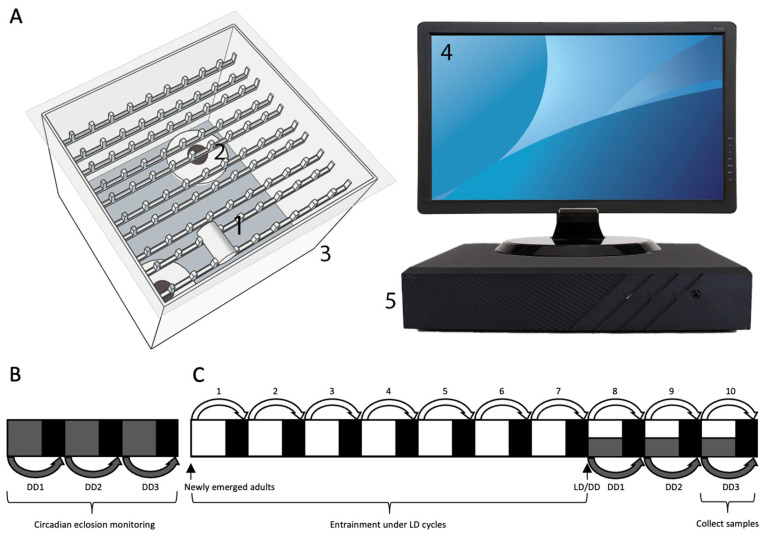
The assay for eclosion rhythm monitoring and adult tissue collection of *S. frugiperda*. (**A**) The real-time eclosion monitoring apparatus for insects. Key components include: 1. Glass tube; 2. Camera; 3. Acrylic stand; 4. Screen, displaying the real-time camera view (powered off during experiments); 5. Video recorder, equipped with a hard drive and control unit for data storage. Cameras are connected to a video recorder via network cables. Granted Chinese patent: ZL202120866706.1. (**B**) Circadian eclosion monitoring assay under the first (DD1), second (DD2), and third (DD3) dark–dark (DD) cycles after entrainment to 14 h light: 10 h dark (L14: D10) cycle. Dark grey and black bars represent subjective day and night, respectively. (**C**) Entraining adults under L14: D10 for seven cycles, followed by sample collection for gene expression analysis after two additional LD or DD cycles. Heads of *S. frugiperda* were collected every 4 h under LD condition and every 3 h under DD3 condition over 24 h.

Eclosion behavior was recorded with a customized apparatus for insect eclosion behavior monitoring (Figure 1A). *S. frugiperda* larvae were raised in summer-like L14: D10 in an incubator (HPG280H, Ningbo Jiangnan Ltd., Ningbo, China) at 26 ± 1 °C and 70 ± 5% RH through pupation. Zeitgeber time 0 (ZT0, 8:30 a.m.) was defined as the time of light onset, and ZT14 (10:30 p.m.) as the time of dark onset. The pupal duration under L14: D10 and DD conditions ranges from 10 to 11.5 days. For acclimation purposes, 7-day-old pupae were gently transferred during the dark phase to numbered glass tubes (2.0 cm outside diameter, 7.0 cm height, 0.1 cm thickness) one hour before the onset of the next LD or DD cycle. Each tube had open ends and was sealed with moistened cotton balls at both ends. All tubes were placed on the apparatus for diel adult eclosion monitoring by an infrared-based camera system under LD condition (Figure 1). Newly emerged adults were removed from the apparatus daily under red light at ZT0. Diel eclosion rhythms were analyzed using the eclosion profile over five consecutive LD cycles.

### 2.3. Circadian Eclosion Behavior Assay Under Constant Darkness

Pupae were transferred from LD to DD at a fixed time point during the late pupal stage. Due to natural inter-individual variation in developmental rates, adult eclosion occurred 1 (DD1), 2 (DD2), or 3 (DD3) days after transfer (Figure 1B). Circadian eclosion behavior assay was performed as described in the LD condition, but under constant darkness. Circadian time 0 (CT0, 8:30 a.m.) is defined as the beginning of the subjective day, and CT14 (10:30 p.m.) is the beginning of the subjective night. Newly emerged adults were removed from the apparatus daily at CT0 under red light. Circadian eclosion rhythms were analyzed using the eclosion profile over three consecutive DD cycles.

### 2.4. Tissue Collection

For RNA extractions of the LD condition, heads from newly emerged female or male adults entrained to ten LD cycles were collected every 4 h over 24 h, with three heads pooled per sample. For RNA extractions of DD condition, heads from female or male adults entrained to seven LD cycles were collected every 3 h over 24 h under red light on the third day of DD (DD3; 10-day-old), with three heads pooled per sample. The samples were stored in an −80 °C fridge until further use.

### 2.5. RNA Isolation and cDNA Synthesis

Three biologically independent head pools, each consisting of three heads, were used for each group defined by sex and sampling time. Total RNA was extracted from these pooled samples using TRIzol^®^ (Invitrogen; Thermo Fisher Scientific, Inc., Waltham, MA, USA). The extracted RNA samples were individually analyzed for quality and quantity using a NanoDrop2000 (Thermo Fisher Scientific, Inc., Waltham, MA, USA). Before reverse transcription, the integrity of each total RNA sample was assessed by electrophoresis in 1% agarose gel. Subsequently, cDNA was synthesized from 100 ng of total RNA in a 20 μL reaction, utilizing the PrimeScript RT reagent kit supplemented with a gDNA Eraser (Takara Bio Inc., Dalian, China).

### 2.6. Gene Expression Analysis

Five target genes, *cyc*, *clk*, *tim*, *per*, and *cry2,* were selected for gene expression analysis using a qRT-PCR assay. Two commonly used reference genes, *EF1-α* and *rpl32*, were assessed for expression stability across sexes. Both exhibited standard deviation values of the cycle threshold (Ct) below 1 (Figure S1), meeting accepted criteria for stable reference genes [54], and neither gene showed consistent expression differences between male and female samples under either photoperiodic (L14: D10) or constant darkness (DD) condition (Figure S2). Based on this, both reference genes were used for normalization to ensure reliable quantification of target gene expression and to minimize the risk of false-positive sex differences in mesor estimates (midline-estimating statistic of rhythm). Primers specific to each gene were designed individually using the Oligo 7 software (Molecular Biology Insights, Inc., Cascade, CO, USA). The synthesis of primers (Table 1) was carried out by GenScript Biotechnology Co., Ltd. (Nanjing, China) and tested as previously described [54]. The qRT-PCR was conducted on an Applied Biosystems^®^ 7500 Fast Real-Time PCR System (Thermo Fisher Scientific, Inc., Waltham, MA, USA) using SYBR Premix Ex Taq (Tli RNaseH Plus; Takara Bio Inc., Dalian, China). The reactions were conducted in a final volume of 20 μL (including 2 μL of a 1/10 dilution of the cDNA template and primers in a final concentration of 200 nM) with the following conditions: an initial 30 s step of 95 °C followed by 40 denaturation cycles at 95 °C for 5 s and primer annealing at 60 °C for 34 s. The *EF1-α* and *rpl32* were used as the reference genes [54], and the 2^−∆∆Ct^ method was applied to evaluate the relative expression levels [55]. Three pooled biological replicates were used for statistical comparisons between groups.

### 2.7. Statistical Analysis

All statistical analyses were performed using SPSS 26. Normality was assessed using the one-sample Kolmogorov–Smirnov (K-S) test for sample sizes greater than 50 and the Shapiro–Wilk test for sample sizes of 50 or fewer (*p* > 0.05). Homogeneity of variances was evaluated using Levene’s test (*p* > 0.05) for all datasets. Data from qRT-PCR analyses met both assumptions and were thus analyzed using parametric tests. Two-way ANOVA was used to test for interaction effects between sex and time under each photoperiod condition. When a marginal or significant interaction was identified, follow-up one-way ANOVA tests were conducted to assess sex differences in gene expression at individual ZT or CT time points. In the absence of such interactions, only main effects were interpreted. Tukey’s HSD test was used for post hoc pairwise comparisons, with adjusted (adj) *p*-values automatically accounting for multiple testing. In contrast, eclosion timing data did not satisfy normality assumptions and were therefore analyzed using non-parametric tests. Pre-defined sex-based differences in eclosion distribution were evaluated using both the two-sample K–S test and the Mann–Whitney U (M–W) test. For exploratory comparisons across DD1–DD3 in males, the K–S and M–W tests were also applied, with Bonferroni-adjusted *P*-values reported. Rhythm characteristics, including mesor, amplitude, and phase, were estimated using the CircaCompare algorithm [56] for each photoperiod-sex combination. In this study, we define “marginal differences” as trends with *P*-values between 0.05 and 0.10, which do not reach conventional significance but may suggest biologically relevant patterns. All data were visualized using GraphPad Prism 10.

## 3. Results

### 3.1. Diel and Circadian Eclosion Rhythms of S. frugiperda Entrained to Summer-like Photoperiod

**Figure 2:**
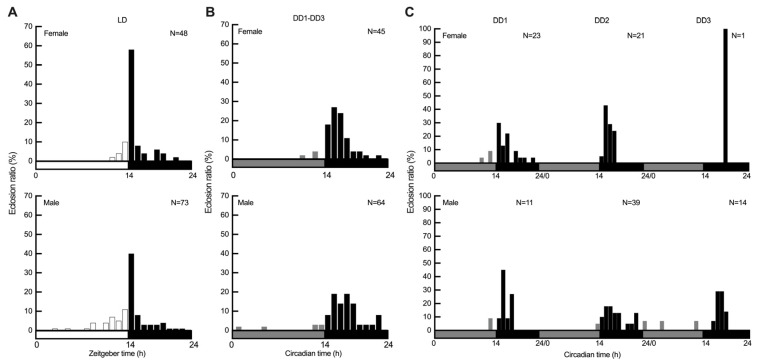
Diel and circadian eclosion rhythms of *S. frugiperda* entrained to L14: D10 and DD. (**A**) Pooled diel eclosion rhythm over five consecutive days of recording under 14 h light: 10 h dark (L14: D10) condition. (**B**) Pooled circadian eclosion rhythm of females and males over three consecutive days of recording under constant darkness (DD). (**C**) Profiles of female and male adult eclosion across the first, second, and third days of constant darkness (DD1, DD2, and DD3 respectively). Data are binned in 1-h intervals. Horizontal bars show objective day (white) and night (black) in panel (**A**) and subjective day (gray) and night (black) in panels (**B**,**C**). N, sample number.

The average eclosion peak under LD condition (pooled data) occurred at the first hour after lights-off (ZT14–ZT15), with 58% of females and 40% of males emerging over 24 h (Figure 2A). The average eclosion peak under DD condition (pooled data) occurred at the second hour after the onset of subjective dark (CT15–CT16), with 27% of females and 19% of males emerging over 24 h. Another average eclosion peak under DD condition was also observed between CT17 and CT18 in male adults (Figure 2B). Under DD condition, the average eclosion peak of *S. frugiperda* exhibited a phase delay of approximately one hour compared to those observed under LD condition. The eclosion peak is sharper and more pronounced under LD condition, whereas in DD, it is more gradual and phase-delayed (Figure 2A,B). Moreover, across successive DD cycles (DD1 to DD3), the eclosion peak progressively shifts later each day (Figure 2C). Females took fewer circadian cycles to complete eclosion, with only one sample emerging in DD3 for the same generation compared to males. Therefore, we excluded the eclosion data for females in DD3 from the subsequent statistical comparison between groups.

### 3.2. Distribution of Pupa Eclosion Between Sexes and Light Regimes

To gain a more detailed understanding of the overall distributions of adult eclosion in *S. frugiperda*, the two-sample K–S test was applied to examine differences in eclosion distributions between light regimes and sexes. A highly significant difference was found when comparing eclosion distributions between pooled LD and DD conditions for both females (*p* < 0.001) and males (*p* < 0.001), indicating a marked shift in timing and pattern under constant darkness, consistent with a circadian-driven free-running period. Additionally, marginal and significant differences were detected between female and male eclosion rhythms under pooled LD (*p* = 0.059) and DD conditions (*p* = 0.019), respectively, suggesting sexually dimorphic circadian regulation of eclosion timing (Figure 2A,B). Due to the limited sample size of females on DD3, statistical comparisons for this group were not performed. Separate analyses of eclosion distributions on DD1 (*p* = 0.362) and DD2 for females and males showed a significant difference between sexes only on DD2 (*p* = 0.017), which aligns with the results from the pooled DD dataset. The manifestation of the differences in the free-running eclosion rhythm of males and females under constant condition may require more than 24 h in *S. frugiperda*. Within-sex comparisons across different DD days revealed only a significant effect between DD1 and DD2 (*p* = 0.010) in females, while no significant differences were found for males (adj *p* ≥ 0.174), suggesting potential differences in the temporal dynamics of circadian regulation between sexes under constant condition.

**Figure 3:**
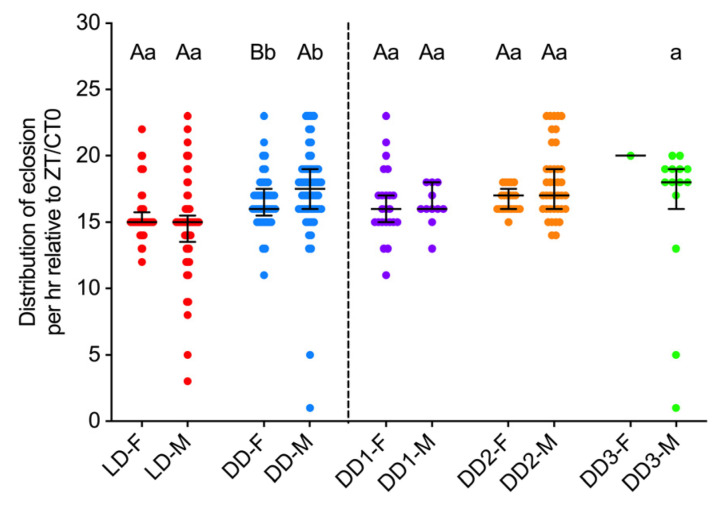
Central tendency comparison for sex differences in eclosion rhythm of *S. frugiperda* under 14 h light: 10 h dark (L14: D10) and constant darkness (DD) conditions. Red, blue, purple, orange, and green dots represent data from five consecutive days under 14 h light: 10 h dark (pooled LD), three consecutive days under constant darkness (pooled DD), the first (DD1), second (DD2), and third (DD3) day of constant darkness, respectively. Solid lines represent the median value, and error bars represent the interquartile range. F, female; M, male. Uppercase and lowercase letters indicated significant differences between sexes for the same photoperiod condition (LD or DD), and between the light regimes for females or males by the Mann–Whitney test at *p* < 0.05, respectively.

Given that the M–W test is sensitive to differences in central tendency (median), while the two-sample K–S test captures overall distributional differences more effectively, the M–W test was further employed to specifically assess differences in the central tendency of adult *S. frugiperda* eclosion per hour relative to ZT0/CT0 between sexes and light regimes. A marginal difference was observed between sexes under LD condition (*p* = 0.095), whereas significant differences were detected between sexes under DD condition (*p* = 0.032). Additionally, highly significant differences were observed when comparing eclosion distributions between pooled LD and pooled DD conditions for both females and males (*p* < 0.001; Figure 3). The results of the M–W test were largely consistent with those of the two-sample K–S test, suggesting that shifts in central tendency contribute to the overall distribution differences. The marginal difference detected by the M–W, as well as the two-sample K–S test under LD conditions may reflect a slight trend in median eclosion times, likely influenced by the higher frequency of male eclosion during the daytime (Figure 2A and Figure 3). We further examined circadian eclosion dynamics separately for DD1 and DD2. Consistent with the two-sample K–S test results, the M–W test revealed no significant difference between sexes on DD1 (*p* = 0.744) and a marginal difference on DD2 (*p* = 0.087). Within-sex comparisons across different DD days revealed no significant differences for both females and males (adj *p* ≥ 0.174), suggesting that the significant difference between DD1 and DD2 for females observed in the two-sample K–S test was primarily due to differences in the distribution shape and spread, rather than in location (Figure 3).

### 3.3. Transcript Expression of Clock Genes in the Heads of S. frugiperda

**Figure 4:**
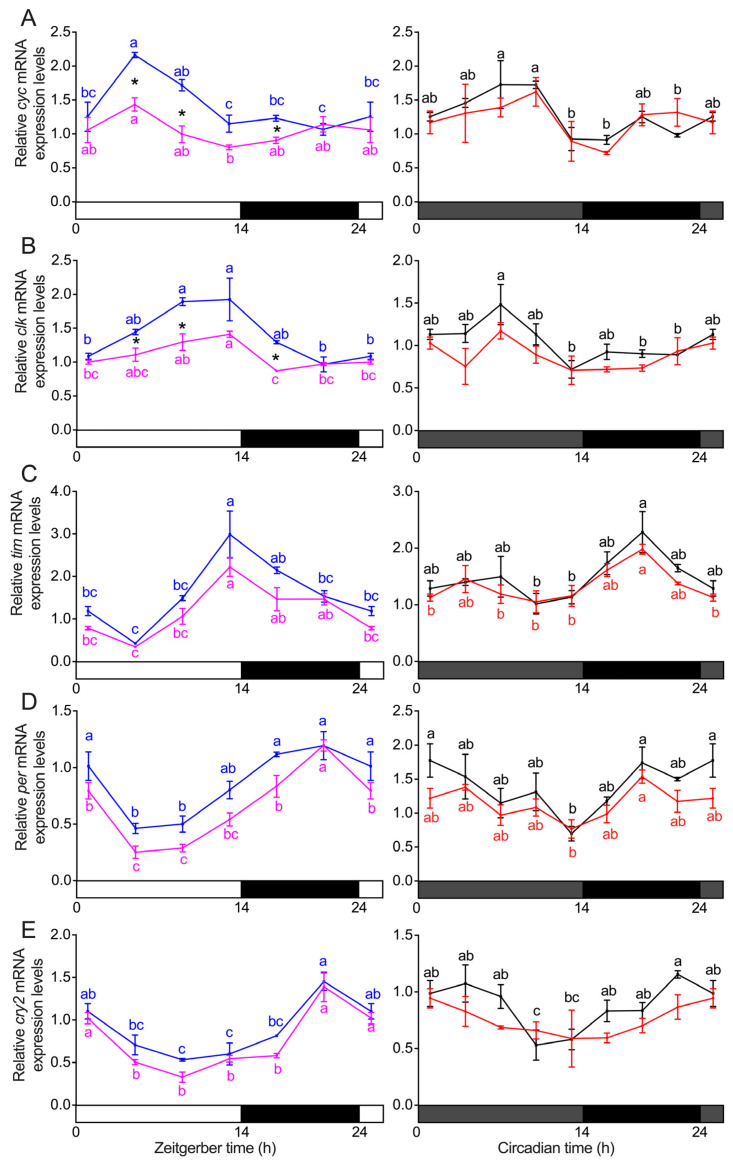
The expression of clock genes of *cyc* (**A**), *clk* (**B**), *tim* (**C**), *per* (**D**), and *cry2* (**E**) in the heads of adults under 14 h light: 10 h dark (LD) and the third day of constant darkness (DD3) after 7 days of adult entrainment to LD. Horizontal bars show objective day (white) and night (black) in the left panels and subjective day (gray) and night (black) in the right panels. Pink and blue lines indicate females (LD-female) and males (LD-male) sampled under LD condition, and red and black lines indicate females (DD3-female) and males (DD3-male) sampled under DD3. Solid lines represent the mean value, and error bars represent the standard error of the mean (SEM). The first and last data points are intentionally duplicated to facilitate visualization of rhythmic patterns across the 24 h cycle. Asterisks indicate significant sex differences at individual ZT or CT time points (*p* < 0.05, one-way ANOVA), conducted only when a significant or marginal interaction was detected in the two-way ANOVA. In the absence of interaction, only main effects were interpreted. Different lowercase letters indicate significant differences among time points within the same sex and light regime (Tukey’s HSD, adj *p* < 0.05). No letters are shown when differences are not statistically significant.

Under the LD condition, significant interactions between sexes and time points were observed for *cyc* (*p* = 0.013; Figure 4A) and a marginal difference for *clk* (*p* = 0.079; Figure 4B) by two-way ANOVA. Significant main effects were found for *tim* (*p* = 0.003; Figure 4C), *per* (*p* < 0.001; Figure 4D), and *cry2* (*p* = 0.011; Figure 4E), with males exhibiting consistently higher expression levels than females across time points for these genes. One-way ANOVA between sexes for the same time point revealed significant sex differences in gene expression levels at ZT5 (*p* = 0.002), ZT9 (*p* = 0.008), and ZT17 (*p* = 0.007) for *cyc* (Figure 4A); ZT5 (*p* = 0.034), ZT9 (*p* = 0.012), and ZT17 (*p* < 0.001) for *clk* (Figure 4B). Under the DD3 condition, no significant interactions between sexes and time points were detected for any of the tested clock genes (*p* ≥ 0.503; Figure 4). Therefore, post hoc comparisons at individual time points were not conducted. Instead, we report the main effects of sex across all time points. Significant main effects of sex were observed for *clk* (*p* = 0.007; Figure 4B), *per* (*p* = 0.017; Figure 4D), and *cry2* (*p* = 0.023; Figure 4E), with males exhibiting consistently higher expression levels than females. In contrast, no significant main effects of sex were detected for *cyc* and *tim* (*p* ≥ 0.171; Figure 4A,C). The presence of rhythmicity was found to be significant for most selected core clock genes in both female and male adults under different light regimes, except female *cyc* and *cry2* under DD3 condition as determined by CircaCompare and confirmed by a post hoc Tukey HSD test at adj *p* < 0.05 (Figure 4; Table 2). Because CircaCompare requires rhythmicity in both groups for valid comparison, parameter estimation was not possible when one sex lacked oscillation. This accounts for the omission of rhythm-related indices for *cyc* and *cry2* under DD conditions in the sex-based comparisons in Table 3.

We further analyzed the mesor (midline-estimating statistic of rhythm), amplitude, and phase differences of selected core clock genes between sexes under LD and DD3 conditions using CircaCompare. Significant and marginal differences in mesor were observed between females and males for *cyc* (−0.38, *p* < 0.001), *clk* (−0.32, *p* < 0.001), *tim* (−0.40, *p* = 0.014), *per* (−0.20, *p* < 0.001), and *cry2* (−0.14, *p* = 0.048) under LD condition. Under DD3 condition, significant mesor differences were found for *clk* (−0.17, *p* = 0.012) and *per* (−0.22, *p* = 0.024). Moreover, significant differences in amplitude were observed between females and males for *clk* (−0.29, *p* = 0.0038) under LD condition. Notably, for phase differences between females (estimated peak at ZT2.96) and males (estimated peak at ZT6.33; Table 3), we found significant differences for *cyc* (−3.37, *p* = 0.044; Table 3) under LD condition; however, the visual inspection of the waveform suggests similar peak phases between sexes, and the CircaCompare model may not capture this perfectly if the gene’s expression pattern deviates from an ideal cosine shape.

We also compared phase differences between LD and DD3 conditions and found significant differences in both females and males for *clk* (females: 5.59, *p* = 0.0069; males: 5.11, *p* < 0.001), *tim* (females: −3.86, *p* = 0.015; males: −4.93, *p* = 0.0021), *per* (females: −3.21, *p* = 0.017; males: −4.07, *p* = 0.0038), and *cry2* (males: −2.42, *p* = 0.021) (Table 3; see Table 4 for estimated peak time hours).

## 4. Discussion

To date, the characteristics of eclosion behavior and the expression patterns of core clock genes in *S. frugiperda* have been rarely investigated [47]. In this study, using a customized monitoring system, we examined potential sex-related differences in both diel and circadian eclosion rhythms, as well as in the expression patterns of core clock genes. Our findings indicate that males and females may differ slightly in certain behavioral outputs of the circadian system, such as eclosion timing, which could be associated with sex-specific life history traits. However, although differences in mesor values of some clock genes were observed between sexes under both light–dark and constant conditions, the overall gene expression profiles and rhythmicity patterns were largely comparable. These results suggest broadly similar circadian regulation across sexes, with limited evidence for consistent or functionally meaningful sex-specific divergence in core clock organization.

The light–dark cycle serves as a strong zeitgeber for diel eclosion rhythm [57,58]. When entrained to a summer-like light–dark (LD) cycle, both female and male *S. frugiperda* exhibited a consistent eclosion peak shortly after lights-off at the light–dark transition, revealing a species-specific window for emergence timing [59,60,61]. Here, although an anticipation of eclosion behavior to light–dark transition was observed before the peak shortly after light-off, the masking effect cannot be excluded when comparing the eclosion pattern with those under DD [62,63]. The observed tendency toward sex-related differences in diel eclosion distribution may reflect variation in clock gene mesor (i.e., average expression level) [25,64], sexually dimorphic masking effects, or a combination of both. However, further investigation is required to clarify their biological significance.

Across successive DD cycles, the eclosion peak shifts later each day, indicating an endogenous free-running period (τ) longer than 24 h, a characteristic of circadian rhythms in the absence of external zeitgebers [14]. Our recent work revealed a *τ* for circadian eclosion of *S. frugiperda* exceeding 24 h with mixed females and males [47]. In this study, we closely examined the potential sexually dimorphic circadian eclosion behavior and observed a consistent τ over 24 h for both females and males. A significant sex difference in the central tendency of pooled eclosion timing was detected, although this difference was not consistently observed across individual days, potentially due to a small sample size. Given that knockout or knockdown of *per* has been shown to induce phase shifts in the circadian rhythms of *Plutella xylostella* and *Nilaparvata lugens* [61,65], the observed tendency toward divergence in emergence timing between sexes may be partially associated with sex-specific variation in *per* transcript expression, although further validation is required to establish any functional relationship. Furthermore, the significant phase shift observed between diel and circadian eclosion profiles may be explained by corresponding shifts in clock gene *tim* and *per* expression between LD and DD conditions in both females and males. Similar associations between eclosion timing and phase shifts in core clock gene expression have been reported in *D. plexippus* [25] and *Delia antiqua* [28].

At the molecular level, while all genes exhibited circadian oscillations under LD and/or DD conditions, sex-related differences were primarily detected in mesor values, rather than in phase or amplitude. These differences may reflect sex-specific modulation of clock gene expression, potentially arising from variation in the number of clock-expressing cells or incomplete dosage compensation of sex-linked genes. However, they provide limited evidence for sexually dimorphic regulation of the circadian system. Under LD condition, the expression patterns of *tim*, *per*, and *cry2* were consistent with those reported by Hänniger et al. for *S. frugiperda* [48], as well as with expression profiles observed in *Agrotis ipsilon* and *D. plexippus* [66]. This similarity suggests that the expression patterns of *tim*, *per*, and *cry2* may be evolutionarily conserved across Lepidoptera. Interestingly, in contrast to our findings, Hänniger et al. (2017) reported that *cyc* and *clk* lacked rhythmic expression under LD in both the rice- and corn-strain FAW [48]. This discrepancy may reflect differences in population background, as the Chinese invasive population likely represents a hybrid lineage. Hybridization between strains could have introduced regulatory variation, potentially resulting in the observed differences in gene rhythmicity, although this hypothesis requires further investigation. CircaCompare analysis revealed that under both LD and DD conditions, the mesor of *clk* and *per* in males was significantly higher than in females, with *cyc*, *tim*, and *cry2* showing significantly higher mesor in males only under LD condition. Hänniger et al. (2017) noted that *cyc*, *clk*, and *per* are located on the Z chromosome in *S. frugiperda*, which follows a ZW sex-determination system (females: ZW; males: ZZ). The presence of two Z chromosomes in males may contribute to an incomplete gene dosage effect [48,67,68], resulting in significantly higher mesor for *cyc*, *clk*, and *per* in males compared to females. As transcriptional activators, the highly expressed *clk* and *cyc* genes may cooperatively promote the expression of downstream genes *tim* and *cry2* [69], potentially contributing to the overall elevated levels of core clock gene transcripts observed in males under LD condition.

Sexual dimorphism in circadian traits has been observed across multiple species [3,4,5,6,7,8,9,10]. Here, the absence of such consistent dimorphism in the invasive Chinese population of *S. frugiperda*, which lacks known strain-level allochrony and likely represents a genetically homogeneous population derived from interstrain hybridization [35,51,70,71], may reflect either the absence of sex-specific circadian variation in the source populations or the maintenance of rhythmic synchrony post-invasion. The latter scenario could be influenced by demographic processes such as genetic bottlenecks or admixture. Further investigation of native populations is necessary to determine whether sex-specific dimorphism in circadian traits existed before invasion. Such studies would provide an essential reference for comparative analyses and offer empirical context for evaluating theoretical models linking sex-specific circadian expression to allochronic speciation [50].

Nonetheless, our results suggest that males and females may exhibit subtle differences in circadian outputs, potentially reflecting an adaptive mechanism for temporal niche partitioning. However, limitations of the present study, including modest sample sizes and the omission of additional core clock genes *vri* and *pdp1*, underscore the need for follow-up research. Future studies integrating other circadian-regulated behaviors, including flight activity [72] and mating [71], under seasonally relevant photoperiods and more ecologically realistic conditions, will be essential to fully understand how the circadian system contributes to behavioral adaptation in a novel migratory context.

## Figures and Tables

**Table 1:** Primers of five clock genes and two reference genes.

Gene	GenBank No.	Sequence (5′ to 3′; F, Forward; R, Reverse)	Purpose
*Qcyc*	XM_035583465.2	F: CAGCAGTCGTCGCATCAACA R: TCGCAGCCAGACATCTCGT	qRT-PCR for *cycle*
*Qclk*	XM_050704408.1	F: GGCACCTCAGGCTACGATTAC R: ATCCACTGCTGCCCTTTTGTT	qRT-PCR for *clock*
*Qtim*	XM_050697432.1	F: GTCTTCATTTGGTTGTTACGGCTA R: CAAGACCAGAAGCGACCTCA	qRT-PCR for *timeless*
*Qper*	XM_035573031.2	F: ATTGTCAGGTTTCAGCGACTTT R: CGATTCTAAGGCACCTGTTACGA	qRT-PCR for *period*
*Qcry2*	XM_050697787.1	F: GGTCGGCATCACTGTCACATC R: CGCTTTGCCACCATTTCGTTCT	qRT-PCR for *cryptochrome 2*
*QEF1-α*	U20139.1	F: CGAAACCGCTATACTATGTCACC R: ATACCAGCCTCGAACTCACC	Reference gene *elongation Factor 1-α*
*Qrpl32*	AF400195.1	F: GGTTCACAGCCGTGTTTCAAT R: ATGGCGGATAAACCTTTTCGTC	Reference gene *ribosomal protein L32*

**Table 2:** Presence of rhythmicity (*p*-value) of five clock genes for different genders and light regimes using the CircaCompare method.

		*cyc*	*clk*	*tim*	*per*	*cry2*
Female	LD	0.0071 **	<0.001 ***	<0.001 ***	<0.001 ***	<0.001 ***
	DD3	ns	0.044 *	0.0058 **	0.010 *	ns
Male	LD	<0.001 ***	<0.001 ***	<0.001 ***	<0.001 ***	<0.001 ***
	DD3	<0.001 ***	0.0010 **	0.0059 **	0.0021 **	<0.001 ***

Note: LD, 14 h light: 10 h dark; DD3, third constant darkness day (DD3) after 7 days of adult entrainment to LD; ns, *p* ≥ 0.05, * *p* < 0.05, ** *p* < 0.01, *** *p* < 0.001.

**Table 3:** Differences in rhythmic parameters across groups using the CircaCompare method.

Gene	Comparison Groups	Mesor Difference (*p*-Value)	Amplitude Difference (*p*-Value)	Phase Difference (*p*-Value)
*cyc*	LD-F vs. LD-M	−0.38 (<0.001 ***)	−0.24 (0.057)	−3.37 (0.044 *)
	LD-F vs. DD3-F	na	na	na
	DD3-F vs. DD3-M	na	na	na
	LD-M vs. DD3-M	na	na	−0.16 (0.90)
				
*clk*	LD-F vs. LD-M	−0.32 (<0.001 ***)	−0.29 (0.0038 **)	−0.64 (0.60)
	LD-F vs. DD3-F	na	na	5.59 (0.0069 **)
	DD3-F vs. DD3-M	−0.17 (0.012 *)	−0.10 (0.27)	−1.12 (0.58)
	LD-M vs. DD3-M	na	na	5.11 (<0.001 ***)
				
*tim*	LD-F vs. LD-M	−0.40 (0.014 *)	−0.27 (0.22)	0.42 (0.68)
	LD-F vs. DD3-F	na	na	−3.86 (0.015 *)
	DD3-F vs. DD3-M	−0.13 (0.24)	−0.094 (0.55)	−0.65 (0.72)
	LD-M vs. DD3-M	na	na	−4.93 (0.0021 **)
				
*per*	LD-F vs. LD-M	−0.20 (<0.001 ***)	0.055 (0.42)	0.45 (0.46)
	LD-F vs. DD3-F	na	na	−3.21 (0.017 *)
	DD3-F vs. DD3-M	−0.22 (0.024 *)	−0.17 (0.22)	−0.41 (0.83)
	LD-M vs. DD3-M	na	na	−4.07 (0.0038 **)
				
*cry2*	LD-F vs. LD-M	−0.14 (0.048 *)	0.037 (0.70)	−0.050 (0.95)
	LD-F vs. DD3-F	na	na	na
	DD3-F vs. DD3-M	na	na	na
	LD-M vs. DD3-M	na	na	−2.42 (0.021 *)

Note: Rhythmicity was found to be significant for all genes in both female and male adults under different light regimes by CircaCompare at *p* < 0.05, except female *cyc* and *cry2* under the third day of constant darkness (DD3) condition. A negative sign indicates that the latter condition is reduced or delayed compared to the former. Data are presented with two significant digits after the decimal point. LD-F or LD-M, heads sampled from female or male adults under 14 h light: 10 h dark (LD); DD3-F or DD3-M: heads sampled from female or male adults under DD3 after 7 days of adult entrainment to LD. na, not applicable, * *p* < 0.05, ** *p* < 0.01, *** *p* < 0.001. For the comparison of group A (former) vs. group B (latter), group B is used as the reference group. Thus, the difference is calculated as A − B (e.g., phase difference = φA − φB).

**Table 4:** Estimated peak time hours of five clock genes for different genders and light regimes using the CircaCompare method.

	LD-F (Hours)	LD-M (Hours)	DD3-F (Hours)	DD3-M (Hours)
*cyc*	2.96	6.33	na	6.49
*clk*	9.87	10.52	4.29	5.40
*tim*	15.42	15.00	19.28	19.93
*per*	20.19	19.74	23.40	23.81
*cry2*	21.98	22.03	na	0.45

Note: LD-F or LD-M, heads sampled from female or male adults under 14 h light: 10 h dark (LD). DD3-F or DD3-M, heads sampled from female or male adults under the third day of constant darkness (DD3) after 7 days of adult entrainment to LD. na, not applicable; used when peak time could not be estimated due to non-significant rhythmicity.

## Data Availability

The original contributions presented in this study are included in the article/Supplementary Material. Further inquiries can be directed to the corresponding author.

## Supplementary Materials

The following supporting information can be downloaded at: https://www.mdpi.com/article/10.3390/insects16070705/s1. Figure S1. Standard deviation (SD) of the reference genes *EF1-α* and *rpl32* across 24 h under LD and DD conditions. Pink and blue lines represent the SD of *EF1-α* and *rpl32*, respectively, under LD conditions, while red and black lines correspond to the same genes under DD3 conditions. Each point denotes the SD value calculated from female and male samples at each time point. A lower SD reflects higher expression stability of the reference gene between sexes at each sampling time point. The first and last data points are intentionally duplicated to facilitate visualization. Figure S2. Geometric mean cycle threshold (Ct) values of two reference genes *EF1-α* and *rpl32* across 24 h under LD and DD conditions. Pink and blue lines represent females and males sampled under LD conditions, while red and black lines represent females and males sampled under DD3. Each point indicates the geometric mean of Ct values at each time point, with error bars representing the standard deviation. Since *EF1-α* and *rpl32* were used jointly for normalization in all qRT-PCR analyses, their geometric mean was calculated to assess the consistency of reference gene expression between sexes across time points. The first and last data points are intentionally duplicated to facilitate visualization.

## Abbreviations

The following abbreviations are used in this manuscript:

L14: D10	14 h light: 10 h dark
DD	Constant darkness
FAW	Fall armyworm
TTFL	Transcriptional–translational feedback loop
LD	Light–dark
ZT	Zeitgeber time
DD1	The first day of constant darkness
DD2	The second day of constant darkness
DD3	The third day of constant darkness
CT	Circadian time
qRT-PCR	Quantitative real-time polymerase chain reaction
K-S	Kolmogorov–Smirnov
M-W	Mann–Whitney

## References

[1] Hardin P.E. (2011). Molecular genetic analysis of circadian timekeeping in *Drosophila*. Adv. Genet..

[2] Reppert S.M., Weaver D.R. (2002). Coordination of circadian timing in mammals. Nature.

[3] Bailey M., Silver R. (2014). Sex differences in circadian timing systems: Implications for disease. Front. Neuroendocrinol..

[4] Duffy J.F., Cain S.W., Chang A.M., Phillips A.J., Munch M.Y., Gronfier C., Wyatt J.K., Dijk D.J., Wright K.P., Czeisler C.A. (2011). Sex difference in the near-24-hour intrinsic period of the human circadian timing system. Proc. Natl. Acad. Sci. USA.

[5] Force E., Sokolowski M.B.C., Suray C., Debernard S., Chatterjee A., Dacher M. (2024). Regulation of feeding dynamics by the circadian clock, light and sex in an adult nocturnal insect. Front. Physiol..

[6] Helfrich-Förster C. (2000). Differential control of morning and evening components in the activity rhythm of *Drosophila melanogaster* sex-specific differences suggest a different quality of activity. J. Biol. Rhythms.

[7] Joye D.A.M., Evans J.A. (2022). Sex differences in daily timekeeping and circadian clock circuits. Semin. Cell Dev. Biol..

[8] Krizo J.A., Mintz E.M. (2015). Sex differences in behavioral circadian rhythms in laboratory rodents. Front. Endocrinol..

[9] Rund S.S., Lee S.J., Bush B.R., Duffield G.E. (2012). Strain- and sex-specific differences in daily flight activity and the circadian clock of *Anopheles Gambiae* mosquitoes. J. Insect Physiol..

[10] Walton J.C., Bumgarner J.R., Nelson R.J. (2022). Sex differences in circadian rhythms. Cold Spring Harbor Perspect. Biol..

[11] Bünning E. (1935). Zur Kenntnis Der Endonomen Tagesrhythmik Bei Insekten Und Bei Pflanzen. Ber. Dtsch. Bot. Ges..

[12] Kalmus H. (1935). Periodizität und autochronie (ideochronie) als zeitregelnde eigenschaffen der organismen. Biol. Gen..

[13] Saunders D.S. (2002). Insect Clocks.

[14] Numata H., Tomioka K. (2023). Insect Chronobiology.

[15] Pittendrigh C.S. (1954). On temperature independence in the clock system controlling emergence time in *Drosophila*. Proc. Natl. Acad. Sci. USA.

[16] Pittendrigh C.S., Skopik S.D. (1970). Circadian systems. V. The driving oscillation and the temporal sequence of development. Proc. Natl. Acad. Sci. USA.

[17] Merlin C., Reppert S.M. (2009). The evolution of circadian clocks in insects. Integr. Comp. Biol..

[18] Mark B., Bustos-González L., Cascallares G., Conejera F., Ewer J. (2021). The circadian clock gates *Drosophila* adult emergence by controlling the timecourse of metamorphosis. Proc. Natl. Acad. Sci. USA.

[19] Wegener C., Amini E., Cavieres-Lepe J., Ewer J. (2024). Neuronal and endocrine mechanisms underlying the circadian gating of eclosion: Insights from *Drosophila*. Curr. Opin. Insect Sci..

[20] Pilorz V., Helfrich-Förster C., Oster H. (2018). The role of the circadian clock system in physiology. Pflügers Arch. Eur. J. Physiol..

[21] Tomioka K., Matsumoto A. (2015). Circadian molecular clockworks in non-model insects. Curr. Opin. Insect Sci..

[22] Dushay M.S., Konopka R.J., Orr D., Greenacre M.L., Kyriacou C.P., Rosbash M., Hall J.C. (1990). Phenotypic and genetic analysis of clock, a new circadian rhythm mutant in *Drosophila melanogaster*. Genetics.

[23] Konopka R.J., Benzer S. (1971). Clock mutants of *Drosophila melanogaster*. Proc. Natl. Acad. Sci. USA.

[24] Sehgal A., Price J.L., Man B., Young M.W. (1994). Loss of circadian behavioral rhythms and per RNA oscillations in the *Drosophila* mutant timeless. Science.

[25] Zhang Y., Markert M.J., Groves S.C., Hardin P.E., Merlin C. (2017). Vertebrate-like CRYPTOCHROME 2 from monarch regulates circadian transcription via independent repression of CLOCK and BMAL1 activity. Proc. Natl. Acad. Sci. USA.

[26] Iiams S.E., Lugena A.B., Zhang Y., Hayden A.N., Merlin C. (2019). Photoperiodic and clock regulation of the vitamin A pathway in the brain mediates seasonal responsiveness in the monarch butterfly. Proc. Natl. Acad. Sci. USA.

[27] Iiams S.E., Wan G.J., Zhang J.W., Lugena A.B., Zhang Y., Hayden A.N., Merlin C. (2024). Loss of functional cryptochrome 1 reduces robustness of 24-hour behavioral rhythms in monarch butterflies. iScience.

[28] Miyazaki Y., Watari Y., Tanaka K., Goto S.G. (2016). Temperature cycle amplitude alters the adult eclosion time and expression pattern of the circadian clock gene period in the onion fly. J. Insect Physiol..

[29] Myers E.M. (2003). The circadian control of eclosion. Chronobiol. Int..

[30] Massy R., Hawkes W.L.S., Doyle T., Troscianko J., Menz M.H.M., Roberts N.W., Chapman J.W., Wotton K.R. (2021). Hoverflies use a time-compensated sun compass to orientate during autumn migration. Proc. Biol. Sci..

[31] Ji J.Y., Liu Y.Q., Zhang L., Cheng Y.X., Stanley D., Jiang X.F. (2023). The clock gene, period, influences migratory flight and reproduction of the oriental armyworm, *Mythimna separata* (Walker). Insect Sci..

[32] Merlin C., Iiams S.E., Lugena A.B. (2020). Monarch butterfly migration moving into the genetic era. Trends Genet..

[33] Merlin C., Liedvogel M. (2019). The genetics and epigenetics of animal migration and orientation: Birds, butterflies and beyond. J. Exp. Biol..

[34] Goergen G., Kumar P.L., Sankung S.B., Togola A., Tamo M. (2016). First report of outbreaks of the fall armyworm *Spodoptera frugiperda* (J E Smith) (Lepidoptera, Noctuidae), a new alien invasive pest in west and central Africa. PLoS ONE.

[35] Kenis M., Benelli G., Biondi A., Calatayud P.A., Day R., Desneux N., Harrison R.D., Kriticos D., Rwomushana I., van den Berg J. (2022). Invasiveness, biology, ecology, and management of the fall armyworm, *Spodoptera frugiperda*. Entomol. Gen..

[36] Tay W.T., Meagher R.L., Czepak C., Groot A.T. (2023). *Spodoptera frugiperda*: Ecology, evolution, and management options of an invasive species. Annu. Rev. Entomol..

[37] Chen H., Wu M.F., Liu J., Shen A.D., Jiang Y.Y., Hu G. (2020). Migratory routes and occurrence divisions of the fall armyworm *Spodoptera frugiperda* in China. J. Plant Prot..

[38] Li X.J., Wu M.F., Ma J., Gao B.Y., Wu Q.L., Chen A.D., Liu J., Jiang Y.Y., Zhai B.P., Early R. (2019). Prediction of migratory routes of the invasive fall armyworm in eastern China using a trajectory analytical approach. Pest Manag. Sci..

[39] Sun X.X., Hu C.X., Jia H.R., Wu Q.L., Shen X.J., Zhao S.Y., Jiang Y.Y., Wu K.M. (2021). Case study on the first immigration of fall armyworm, *Spodoptera frugiperda* invading into China. J. Integr. Agric..

[40] Huang Y.Y., Lv H., Dong Y.Y., Huang W.J., Hu G., Liu Y., Chen H., Geng Y., Bai J., Guo P. (2022). Mapping the spatio-temporal distribution of fall armyworm in China by coupling multi-factors. Remote Sens..

[41] Jiang C.X., Zhang X.Y., Wu J., Feng C.H., Ma L., Hu G., Li Q. (2022). The source areas and migratory pathways of the fall armyworm *Spodoptera frugiperda* (Smith) in Sichuan province, China. Insects.

[42] Wu Q.L., Jiang Y.Y., Liu J., Hu G., Wu K.M. (2021). Trajectory modeling revealed a southwest-northeast migration corridor for fall armyworm *Spodoptera frugiperda* (Lepidoptera: Noctuidae) emerging from the north China plain. Insect Sci..

[43] Zhang X.Y., Huang L., Liu J., Zhang H.B., Qiu K., Lu F., Hu G. (2023). Migration dynamics of fall armyworm *Spodoptera frugiperda* (Smith) in the Yangtze river delta. Insects.

[44] Chen H., Wan G.J., Li J.C., Ma Y.B., Reynolds D.R., Dreyer D., Warrant E.J., Chapman J.W., Hu G. (2023). Adaptive migratory orientation of an invasive pest on a new continent. iScience.

[45] Denlinger D.L., Hahn D.A., Merlin C., Holzapfel C.M., Bradshaw W.E. (2017). Keeping time without a Spine: What can the insect clock teach us about seasonal adaptation?. Phil. Trans. R. Soc. B.

[46] Jin M.H., Liu B., Zheng W.G., Liu C.H., Liu Z.X., He Y., Li X., Wu C., Wang P., Liu K.Y. (2023). Chromosome-level genome of black cutworm provides novel insights into polyphagy and seasonal migration in insects. BMC Biol..

[47] Lv C.N., Huang X., Wang W.T., He Y.K., Hu G., Pan W.D., Chen F.J., Wan G.J. (2023). The circadian eclosion rhythm of *Spodoptera frugiperda* in response to photoperiod cues. Chin. J. Appl. Entomol..

[48] Hänniger S., Dumas P., Schöfl G., Gebauer-Jung S., Vogel H., Unbehend M., Heckel D.G., Groot A.T. (2017). Genetic basis of allochronic differentiation in the fall armyworm. BMC Evol. Biol..

[49] Tessnow A.E., Nagoshi R.N., Meagher R.L., Gilligan T.M., Sadd B.M., Carriere Y., Davis H.N., Fleischer S.J., Richers K., Palumbo J.C. (2025). Genomic patterns of strain-specific genetic structure, linkage, and selection across fall armyworm populations. BMC Genom..

[50] van Doorn G.S., Schepers J., Hut R.A., Groot A.T. (2024). Sex-specific expression of circadian rhythms enables allochronic speciation. Evol. Lett..

[51] Wang X., Du Z.Y., Duan Y.G., Liu S.L., Liu J., Li B.Y., Ma L., Wu Y.F., Tian L., Song F. (2024). Population genomics analyses reveal the role of hybridization in the rapid invasion of fall armyworm. J. Adv. Res..

[52] Wan G., Sword G.A., Du J., Huang Q., Chen W., Warrant E. (2025). Editorial: Magnetobiology and chronobiology: New opportunities for smart phytoprotection. Front. Plant. Sci..

[53] Li Z.Y., Dai Q.X., Kuang Z.L., Liang M.R., Wang L., Lu Y.Y., Chen K. (2019). Effects of three artificial diets on development and reproduction of the fall armyworm *Spodoptera frugiperda* (J.E. Smith). J. Environ. Entomol..

[54] Zhang Y., Zeng L., Wei Y.J., Zhang M., Pan W.D., Sword G.A., Yang F., Chen F.J., Wan G.J. (2022). Reliable reference genes for gene expression analyses under the hypomagnetic field in a migratory insect. Front. Physiol..

[55] Livak K.J., Schmittgen T.D. (2001). Analysis of relative gene expression data using real-time quantitative PCR and the 2(-delta delta C(T)) method. Methods.

[56] Parsons R., Parsons R., Garner N., Oster H., Rawashdeh O. (2019). CircaCompare: A method to estimate and statistically support differences in mesor, amplitude and phase, between circadian rhythms. Bioinformatics.

[57] Stanewsky R. (2002). Clock mechanisms in *Drosophila*. Cell Tissue Res..

[58] Merlin C. (2009). Lepidopteran Circadian Clocks: From Molecules to Behavior. Molecular Biology and Genetics of the Lepidoptera.

[59] Bertossa R.C., van Dijk J., Beersma D.G., Beukeboom L.W. (2010). Circadian rhythms of adult emergence and activity but not eclosion in males of the parasitic wasp *Nasonia vitripennis*. J. Insect Physiol..

[60] Nartey M.A., Sun X., Qin S., Hou C.X., Li M.W. (2021). CRISPR/Cas9-based knockout reveals that the clock gene timeless is indispensable for regulating circadian behavioral rhythms in *Bombyx mori*. Insect Sci..

[61] Wang D.F., Chen J., Yuan Y., Yu L.Q., Yang G., Chen W.F. (2023). CRISPR/Cas9-mediated knockout of *Period* reveals its function in the circadian rhythms of the diamondback moth *Plutella xylostella*. Insect Sci..

[62] Beer K., Helfrich-Förster C. (2020). Model and non-model insects in chronobiology. Front. Behav. Neurosci..

[63] Bidell D., Feige N.D., Triphan T., Muller C., Pauls D., Helfrich-Förster C., Selcho M. (2024). Photoreceptors for immediate effects of light on circadian behavior. iScience.

[64] Malekera M.J., Acharya R., Mostafiz M.M., Hwang H.S., Bhusal N., Lee K.Y. (2022). Temperature-dependent development models describing the effects of temperature on the development of the fall armyworm *Spodoptera frugiperda* (J. E. Smith) (Lepidoptera: Noctuidae). Insects.

[65] Wei Q., He J.C., Wang W.X., Lai F.X., Wan P.J., Fu Q. (2025). Role of the clock gene *Period* in regulating circadian rhythm of courtship vibrations in *Nilaparvata lugens*. Insect Biochem. Mol. Biol..

[66] Li G.Y., Cui Q., Zheng S.R., Zhang K.X., Wang Y.H., Zhan S., Fang G.Q. (2025). Molecular basis of circadian rhythm divergence between diurnal and nocturnal Lepidoperans. iScience.

[67] Xie X., Zhang Y., Peng H., Deng Z. (2025). Sex chromosome dosage compensation in insects. Insects.

[68] Dayton J.N., Dopman E.B. (2024). The *period* gene alters daily and seasonal timing in *Ostrinia nubilalis*. bioRxiv.

[69] Brady D., Saviane A., Cappellozza S., Sandrelli F. (2021). The circadian clock in Lepidoptera. Front. Physiol..

[70] Zhang L., Li Z.Y., Peng Y., Liang X.Y., Wilson K., Chipabika G., Karangwa P., Uzayisenga B., Mensah B.A., Kachigamba D.L. (2023). Global genomic signature reveals the evolution of fall armyworm in the eastern hemisphere. Mol. Ecol..

[71] Zhang L.Y., Wang F., Wan X.S., Xu J., Ye H. (2022). Reproductive behavior and circadian rhythms of *Spodoptera frugiperda*. J. Environ. Entomol..

[72] He L.M., Ge S.S., Zhang H.W., He W., Yan R., Wu K.M. (2021). Photoregime affects development, reproduction, and flight performance of the invasive fall armyworm (Lepidoptera: Noctuidae) in China. Environ. Entomol..

