# Supplementary material for: Comparative Analysis of Diel and Circadian Eclosion Rhythms and Clock Gene Expression Between Sexes in the Migratory Moth Spodoptera frugiperda
Source: Insects. 2025 Jul 9;16(7):705. doi: 10.3390/insects16070705 (PMC12296149; doi:10.3390/insects16070705)
Supplement: Supplementary file 1 [file insects-16-00705-s001.zip › insects-3658567-supplementary.pdf]

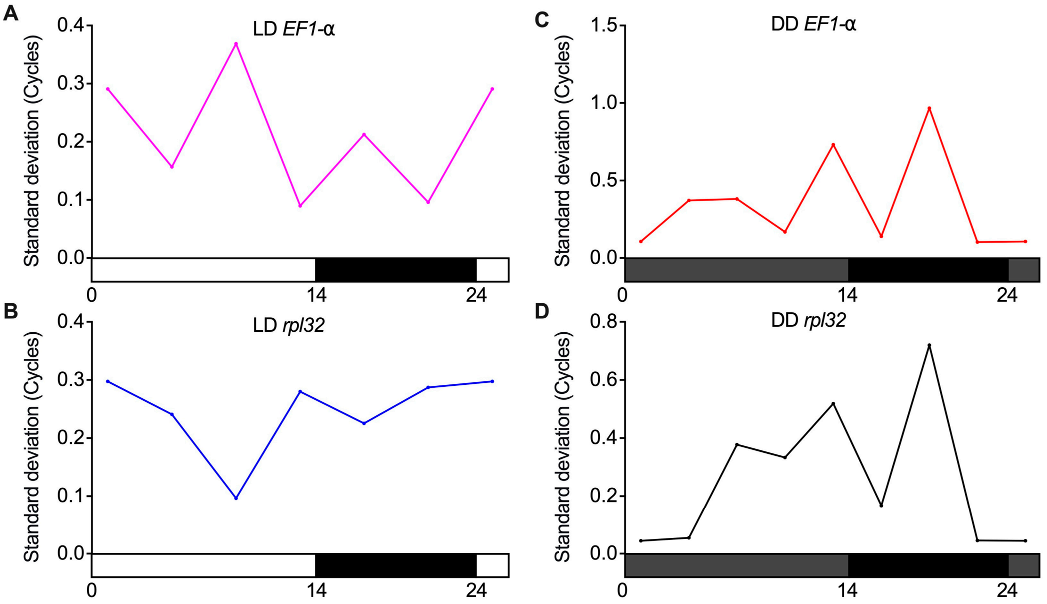

Figure S1. Standard deviation (SD) of the reference genes *EF1-α* and *rpl32* across 24 hours under LD and DD conditions. Pink and blue lines represent the SD of *EF1-α* and *rpl32*, respectively, under LD conditions, while red and black lines correspond to the same genes under DD3 conditions. Each point denotes the SD value calculated from female and male samples at each time point. A lower SD reflects higher expression stability of the reference gene between sexes at each sampling time point. The first and last data points are intentionally duplicated to facilitate visualization.

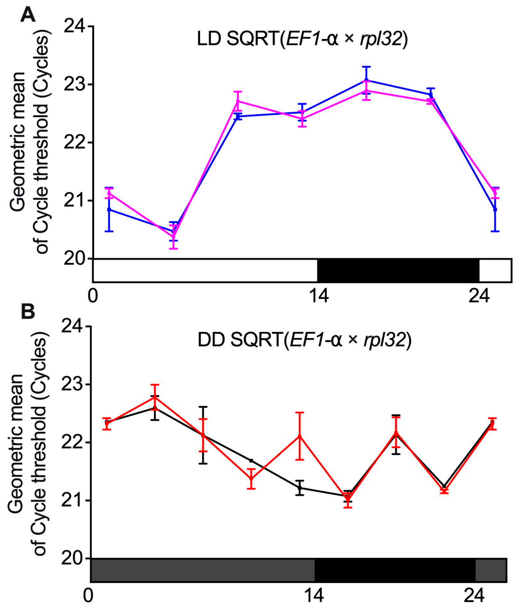

Figure S2. Geometric mean cycle threshold (Ct) values of two reference genes *EF1-α* and *rpl32* across 24 hours under LD and DD conditions. Pink and blue lines represent females and males sampled under LD conditions, while red and black lines represent females and males sampled under DD3. Each point indicates the geometric mean of Ct values at each time point, with error bars representing the standard deviation. Since *EF1-α* and *rpl32* were used jointly for normalization in all qRT-PCR analyses, their geometric mean was

calculated to assess the consistency of reference gene expression between sexes across time points. The first and last data points are intentionally duplicated to facilitate visualization.
